# ANTI-REFLUX PROCEDURES AFTER ROUX-EN-Y GASTRIC BYPASS

**DOI:** 10.1590/0102-672020210002e1614

**Published:** 2021-12-17

**Authors:** David MOTOLA, Ibrahim M. ZEINI, Rena C. MOON, Muhammad GHANEM, Andre F. TEIXEIRA, Muhammad A. JAWAD

**Affiliations:** 1 Orlando Health Weight Loss and Bariatric Surgery Institute, Orlando Health, Orlando, FL, USA

**Keywords:** Obesity, Gastroesophageal Reflux, Gastric Bypass, Roux-en-Y Anastomosis, Obesidade, Refluxo gastroesofágico, Derivação gástrica, Anastomose em Y-Roux

## Abstract

**Background:**

Roux-en-Y gastric bypass (RYGB) has been the choice of bariatric procedure for patients with symptomatic reflux - and is known to be effective in reducing the need for anti-reflux medication postoperatively. However, a small number of RYGB patients can still develop severe reflux symptoms that require a surgical intervention.

**Aim:**

To examine and describe the patient population that requires an anti-reflux procedure after RYGB evaluating demographics, characteristics, symptoms and diagnosis

**Methods:**

A retrospective chart review was performed on 32 patients who underwent a hiatal hernia repair and/or Nissen fundoplication after RYGB Jul 1^st^, 2014 and Dec 31^st^, 2019. Patients were identified using the MBSAQIP database and their electronic medical records were reviewed.

**Results:**

Most patients were female (n=29, 90.6%). The mean age was 52.8 years and the mean body mass index (BMI) was 34.1 kg/m^2^ at the time of anti-reflux procedure. Patients underwent the anti-reflux procedure at a mean of 7.9 years after the RYGB procedure. The mean percentage of excess BMI loss during the time between RYGB and anti-reflux procedure was 63.4%.

**Conclusions:**

Female patients with a significant weight loss may develop a severe reflux symptoms years after RYGB. Complaints of reflux after RYGB should not be overlooked. Careful follow-up and appropriate treatment (including surgical intervention) is needed for this population.

## RESUMO

**Racional:**

O bypass gástrico em Y-de-Roux (RYGB) tem sido o procedimento bariátrico de escolha para pacientes com refluxo sintomático - e é conhecido por ser eficaz na redução da necessidade de medicação anti-refluxo no pós-operatório. No entanto, um pequeno número de pacientes com RYGB ainda pode desenvolver sintomas de refluxo graves que requerem uma intervenção cirúrgica.

**Objetivo:**

Examinar e descrever a população de pacientes que requer procedimento anti-refluxo após RYGB avaliando dados demográficos, características, sintomas e diagnóstico.

**Métodos:**

Revisão retrospectiva de prontuários foi realizada em 32 pacientes submetidos a hérnia hiatal e / ou fundoplicatura Nissen após RYGB em 1º de julho de 2014 a 31 de dezembro de 2019. Os pacientes foram identificados por meio do banco de dados MBSAQIP e seus prontuários eletrônicos foram revisados.

**Resultados:**

A maioria dos pacientes era do sexo feminino (n = 29 - 90,6%). A média de idade foi de 52,8 anos e o índice de massa corporea (IMC) médio de 34,1 kg / m2 na época do procedimento anti-refluxo. Os pacientes foram submetidos ao procedimento anti-refluxo em média 7,9 anos após o procedimento do BGYR. A porcentagem média de perda do excesso de IMC durante o tempo entre o BGYR e o procedimento anti-refluxo foi de 63,4%.

**Conclusões:**

Pacientes do sexo feminino com perda de peso significativa podem desenvolver sintomas graves de refluxo anos após o BGYR. Sintomas de refluxo após RYGB não devem ser negligenciadas. Acompanhamento cuidadoso e tratamento adequado (incluindo intervenção cirúrgica) são necessários para essa população.

## INTRODUCTION

In recent years, laparoscopic sleeve gastrectomy (LSG) became the most commonly performed bariatric procedure according to American Society for Metabolic and Bariatric Surgery^11^. Nevertheless, Roux-en-Y gastric bypass (RYGB) still constituted 17% of all bariatric procedures in 2018^11^. Up to 70% of preoperative bariatric patients suffer from gastroesophageal reflux disease (GERD) symptoms and between 5-50% of obese individuals are reported to have hiatal hernia^2^ ^,^ ^4^. Obese individuals are more prone to hiatal hernias and esophagitis secondary to unique changes in physiology as it pertains to increased intra-abdominal pressure^7^. They are more than four times as likely to have hiatal hernias than normal weight patients^26^. Several studies reviewed preoperative workup for these entities and ways to manage them at the time of initial bypass^2^ ^,^ ^13^ ^,^ ^24^.

The RYGB is a durable operation that has been demonstrated to be both effective for weight loss and reducing the need for anti-reflux medication postoperatively^1^ ^,^ ^3^ ^,^ ^24^. However, a substantial number of patients, up to 22%, who undergo successful RYGB continued to complain of heartburn postoperatively^8^. Not many studies documented the prevalence of hiatal hernia following bariatric surgery nor the degree to which physiologic reflux develops following RYGB^12^. Pallati et al.^23^ found that improvement in GERD score was significantly higher in RYGB when compared to sleeve gastrectomy. Nevertheless, RYGB is not free from postoperative reflux. Several case studies report findings of hiatal hernia causing symptoms ranging from abdominal pain to failure to thrive after a RYGB^12, 6,16,18^. At our center, we observed RYGB patients complaining of severe GERD or dysphagia years after the primary procedure that required a surgical intervention. 

This has the objective to review 32 patients who underwent an anti-reflux procedure such as hiatal hernia repair and/or Nissen fundoplication after RYGB evaluating the demographics of patients at the time of anti-reflux procedures after Roux-en-Y gastric bypass; characteristics of patients undergoing anti-reflux procedure after Roux-en-Y gastric bypass; presenting symptoms and diagnosis method for patients undergoing anti-reflux procedure after Roux-en-Y gastric bypass

## METHODS

After Institutional Review Board approval and following Health Insurance Portability and Accountability Act Guidelines, all patients who underwent hiatal hernia repair and/or Nissen fundoplication after RYGB between July 1^st^, 2014 and December 31^st^, 2019 were identified using the MBSAQIP (Metabolic and Bariatric Surgery Accreditation and Quality Improvement Program) database. A total of 32 patients were identified. Electronic medical records of these patients were retrospectively reviewed. Presenting symptoms for the anti-reflux procedures included reflux, dysphagia, nausea and vomiting, and abdominal pain. Most patients were diagnosed using the upper gastrointestinal series (UGI) or both UGI and upper endoscopy. Some patients were diagnosed with hiatal hernia intra-operatively at the index RYGB.

### Surgical technique

The procedures were performed at a single center by two experienced bariatric surgeons with over 10,700 procedures. Roux-en-Y gastric bypass was completed via robotic-assisted laparoscopy. A 15-30 ml gastric pouch was first created laparoscopically by creating a window between the lesser omentum and stomach along the lesser curvature, with preservation of the nerve of Latarjet. A 34-Fr bougie sized Edlich tube (Covidien, Mansfield, MA) was passed orally into the stomach and used to aid size determination of the pouch. Approximately five 3.5 mm laparoscopic staplers were used to transect the stomach along the lesser curvature up to the esophagogastric angle (His). The staple lines were not oversewn and were buttressed with Surgicel Nu-knit (Johnson and Johnson, Somerville, NJ, USA). The da Vinci assisted robotic system was docked to the three 8 mm trocars. The greater omentum was split using a harmonic scalpel to allow the omentum and transverse colon to retract superiorly. The Roux limb was created by following the jejunum 40 cm distal to the duodenojejunal ligament (Treitz) and transecting the jejunum with a linear stapler. The side-to-side jejunojejunostomy was created approximately at 75 cm for patients with BMI less than 45 kg/m^2^, at 100 cm for BMI between 45 and 55 kg/m^2^, and at 150 cm for BMI over 55 kg/m^2^ distal to the transection point along the efferent limb. A single 2.5 mm laparoscopic stapler was fired, and the enteric defect was then closed using a two-layer 2-0 Polysorb full cm for BMI over thickness running suture followed by a running Lambert suture. An antecolic, antegastric gastrojejunostomy was created by bringing the proximal efferent limb to the gastric pouch and performing a two-layer hand-sewn anastomosis. A running 2-0 Polysorb suture was used to approximate the efferent limb mesentery to the transverse mesocolon, obliterating the Peterson’s defect. An anterior gastrostomy was created in the distal gastric body, and a gastrostomy tube was placed using a 24 F Foley catheter through the left upper quadrant trocar. The anastomoses were then tested under air insufflation and subsequently with methylene blue injection. The da Vinci assisted robotic system was un-docked, and the port sites were closed using standard closure techniques as described in our previous publication^21^.

The hiatal hernia operation is typically divided into three steps. First, dissection and reduction of the hernia sac and stomach into the abdomen, then closure of the crural/hiatal defect, and finally creation of the fundoplication. The fundoplication is done by mobilizing the greater curve of the stomach remnant and pass it behind the esophagus to do the Nissen fundoplication using non absorbable suture. Techniques differ, but these basic steps are common to all hiatal hernia repairs. Veress needle was inserted in midclavicular line on left side and tested with saline drop. Abdomen is insufflated with CO_2_. An 8 mm trocar was used to insert 5 mm scope. Visualization of abdominal cavity to identify any abnormalities. Left lobe of the liver is retracted anteriorily by attaching allis grasper to the diaphragm. After docking the da Vinci, a vessel sealer, the bipolar, and the cadiere grasper are utilized. Next the gastrohepatic ligament is taken down up to the right crus and continuing dissection of the peritoneum over to the left crus. Then a short gastric vessel is taken down along the greater curvature until it is fully mobilized. Then the hiatal hernia is dissected away from the esophagus starting at the right crus up to the confluence of the right and left crus ensuring not to injure the anterior or posterior vagus nerves. We then create a window posteriorly to the esophagus dissecting the hiatal hernia sac free of the left crus. Hiatus is then closed with a #0 V-Lock suture in a running fashion ensuring to lock the last suture. Then the superior portion of the greater curvature is pulled posteriorly behind the esophagus to the lesser curvature and created the Nissen fundoplication. A 40 F bougie catheter is passed into the stomach and then create the Nissen fundoplication using 2-0 Vicryl suture. Three interrupted stitches are placed to complete the wrap. Then the bougie is removed and the da Vinci robot is undocked. The trocars are removed under directed vision and the abdomen desufflated. The trocar sites are closed with 2-0 Vicryl and dermabond. 

### Statistical analysis

Descriptive statistics were used for data presentation. All data were demonstrated as frequency or mean and standard deviation unless otherwise noted. Median (lower quartile, upper quartile) were used when the normality assumption was violated. All statistical analyses were performed using SAS University Edition (SAS Institute, Cary, NC).

## RESULTS

Patient characteristics included 90.6% female with a mean age of 52.8 years old and a mean BMI of 34.1 kg/m^2^ at the time of anti-reflux procedures (Table 1). Of these patients, 43.8% had hypertension, 6.3% had diabetes mellitus, 15.6% had sleep apnea, and 28.1% had hyperlipidemia. Most patients underwent hiatal hernia repair (75.0%), while four (12.5%) underwent Nissen fundoplication and another four (12.5%) both procedures. Median length of hospital stay was one day. 

**Table 1:** Demographics of patients at the time of anti-reflux procedures after Roux-en-Y gastric bypass

Characteristics	Number of patients (n=32)
Female, n(%)	29 (90.6)
Age (years), mean(std)	52.8 (10.3)
BMI (kg/m^2^), mean(std)	34.1 (7.7)
Comorbidities, n (%) Hypertension Diabetes mellitus Sleep apnea Hyperlipidemia	14 (43.8) 2 (6.3) 5 (15.6) 9 (28.1)
Procedure, n(%) Hiatal hernia repair Nissen fundoplication Both	24 (75.0) 4 (12.5) 4 (12.5)
Length of hospital stay (days), median(q1, q3)	1 (1, 2)

BMI: body mass index

RYGB: Roux-en-Y gastric bypass

These patients underwent the anti-reflux procedure at a mean of eight years after the RYGB (Table 2). Where available, the mean BMI of these patients at the time of RYGB was 46.5 kg/m^2^, and the median percentage of excess BMI loss of these patients was 63.3%. Two patients underwent hiatal hernia repair at the time of index RYGB. Three underwent the index RYGB procedure as a conversion - 2 from gastric banding and one from sleeve gastrectomy. For patients undergoing RYGB and anti-reflux procedure at the same time, 12.5% of patients underwent endoscopic dilation of the gastrojejunostomy. 

**Table 2:** Characteristics of patients undergoing anti-reflux procedure after Roux-en-Y gastric bypass

Characteristics	N=32
Time from RYGB to anti-reflux procedure (years), mean(std)^a^	8.0 (4.5)
BMI at RYGB (kg/m^2^), mean(std)^a^	46.5 (8.8)
EBMIL from RYGB to anti-reflux procedure (%), median(q1, q3)^a^	63.3 (44.3, 83.2)
Hiatal Hernia Repair at the time of RYGB, n(%)	2 (6.3)
Procedure prior to RYGB, n(%) Gastric banding Sleeve gastrectomy	2 (6.3) 1 (3.1)
Procedures prior to the anti-reflux procedure, n(%) None Endoscopic dilation of the gastrojejunostomy Laparoscopic internal hernia repair Laparoscopic revision of the gastrojejunostomy Endoscopic sclerotherapy	24 (75.0) 4 (12.5) 2 (6.3) 1 (3.1) 1 (3.1)

^a^ When data were available (n=27 for weight and n=30 for time from RYGB)

Presenting symptoms for the anti-reflux procedures included reflux (n=29), dysphagia (n=18), nausea and vomiting (n=16), and abdominal pain (n=14, Table 3). Most patients were diagnosed using the upper gastrointestinal series (UGI, n=14) or both UGI and upper endoscopy (n=14). Two patients had their hiatal hernia diagnosed intra-operatively at the index RYGB.

**Table 3:** Presenting symptoms and diagnosis method for patients undergoing anti-reflux procedure after Roux-en-Y gastric bypass

Characteristics	N=32
Presenting symptoms, n(%) Reflux Dysphagia Nausea and vomiting Weight gain Abdominal pain Dumping	29 (90.6) 18 (56.3) 16 (50.0) 16 (50.0) 14 (43.8) 3 (9.4)
Diagnosis method for reflux, n(%) Upper endoscopy Upper gastrointestinal series Both Other (identified visually during the procedure)	2 (6.3) 14 (43.8) 14 (43.8) 2 (6.3)

At a mean follow up of 18 months (1-69), 26 patients remained symptom free. Four required hiatal hernia re-operations. One developed an anastomotic ulcer.

## DISCUSSION

Our study shows that some patients can develop reflux symptoms after RYGB severe enough to require a surgical intervention. Reflux symptoms are generally considered as a common adverse event after LSG procedures; conversion to a RYGB is one of the options provided to patients with severe reflux symptoms after LSG^9^. Preoperatively, patients with reflux symptoms are deterred from LSG and recommended to undergo RYGB as well^5^ ^,^ ^15^. The mechanism behind this rationale include low-pressure system, fewer acid-producing parietal cells in the smaller pouch, and longer alimentary limb preventing the return of biliopancreatic content^20^. Several large studies in the United States showed that 50-60% of RYGB patients had decreased reflux symptoms within one-year following the procedure^10^ ^,^ ^23^ ^,^ ^25^. 

Nevertheless, in a recent large population-based cohort study, Holmberg et al.^17^ suggested that the effectiveness of RYGB procedure on reflux symptoms may have been overstated. In their study of 2454 RYGB patients with pre-operative reflux symptoms, the prevalence of reflux requiring anti-reflux medication was approximately 68% during five years after the procedure.

They also pointed out that not many studies have demonstrated the long-term effect of RYGB on resolving reflux symptoms^23^. Of those that did, the sample sizes were small and the assessment of reflux symptoms could have introduced uncertainty of the internal validity^14^ ^,^ ^19^ ^,^ ^22^. 

We think it is important to note the timing of developing and/or resolving reflux symptoms. In a recent review article, Crawford et al.^9^ indicated that most reflux symptoms after LSG were observed within one-year after surgery. Considering that approximately 50% of patients had resolution of reflux symptoms within one-year after RYGB^17^, practicing surgeons may have the impression that RYGB is much superior than LSG in terms of resolving reflux symptoms. However, at our practice, we have observed many patients complaining of reflux symptoms years after RYGB. In fact, average time from RYGB to an anti-reflux procedure was eight years in this study - highlighting the necessity of long-term follow-up on reflux symptoms after RYGB. 

Holmberg et al.^17^ identified that the use of a higher dose of anti-reflux medication prior to the RYGB procedure was the strongest indicator of persistent reflux symptoms after the procedure. Based on this report and our study, we believe RYGB should not be considered as a ‘cure’ for reflux symptoms in the bariatric population - and caution should be taken in absentmindedly proceeding with the procedure among patients taking high dose of anti-reflux medication preoperatively.

It is also important to note that only 6.3% (n=2) of these patients underwent hiatal hernia repair at the time of RYGB. This may indicate that surgeons should be more judicious in seeking out and aggressively treating hiatal hernias at the time of RYGB. As the aforementioned research has indicated, obese individuals are more likely to develop hiatal hernias even before undergoing bypass^7^. Although hiatal hernia repair at the time of RYGB may be more technically challenging - obesity impairs reduction of the hiatal hernia sac, pillar identification and dissection, and tension-free closure of the hiatus^3^ - patients may enjoy a better quality of life and avoid further surgical intervention for anti-reflux procedures. 

This study demonstrates that patients experiencing severe reflux symptoms years after RYGB procedure can be safely and effectively treated with an anti-reflux procedure. Most of our patients (81%) were free of symptoms at a mean follow-up of 18 months. 

There are limitations in this research. First, this was a retrospective study with a small number of patients. We were not able to identify the true incidence of reflux symptoms in the RYGB population since the percentage of long-term follow-up was low and patients could have sought care from a different provider. Second, we only identified the timing of anti-reflux procedure when the symptoms could have started months and years prior. Only those who had symptoms severe enough to undergo an invasive intervention were captured in this study. Regardless, this study is one of few studies demonstrating the need for anti-reflux procedures after RYGB. We believe this adds an important viewpoint to practicing surgeons when they encounter patients with severe reflux symptoms before and after RYGB. A larger prospective, randomized study with longer follow-up is needed to assess the effectiveness of RYGB on reflux symptom.

## CONCLUSIONS

Patients may develop severe reflux symptoms after RYGB. These patients may be safely and effectively treated with anti-reflux procedures. RYGB should not be considered the cure-all for reflux symptoms in the bariatric population. Further, prospective study of the true incidence of reflux and objective measures of symptoms early on may allow for better symptom management. Female patients with a significant weight loss may develop a severe reflux symptoms years after RYGB. Complaints of reflux after RYGB should not be overlooked. Careful follow-up and appropriate treatment (including surgical intervention) is needed for this population.
